# Novel *TRPV3* loss-of-function mutation in Olmsted syndrome with attenuated phenotype

**DOI:** 10.1016/j.jdcr.2024.05.013

**Published:** 2024-05-17

**Authors:** Travis Frantz, David Kirwin, Angela Crotty, Willis Lyford

**Affiliations:** Department of Dermatology, Naval Medical Readiness Training Center San Diego, San Diego, California

**Keywords:** genodermatosis, Olmsted syndrome, palmoplantar keratoderma, transient receptor potential vanilloid-3

## Introduction

Olmsted syndrome (OS) is a rare genodermatosis first described in 1927.^1^ Since then, approximately 85 cases have been reported. OS does not appear to have an ethnic predilection but is more common in males. Thus far, gain-of-function mutations in transient receptor potential vanilloid-3 (*TRPV3*) and mutations in membrane-bound transcription factor protease site 2 (*MBTPS2*) have been associated with inheritance of OS in an autosomal and X-linked recessive manner, respectively.^2^^,^^3^ Mutations in *PERP* (*TP53* apoptosis effector) have also been reported to cause OS.^4^ To our knowledge, we present the first known case of a loss-of-function mutation in the *TRPV3* gene with an associated mild phenotypic presentation of features seen in OS.

## Case report

**Fig 1:**
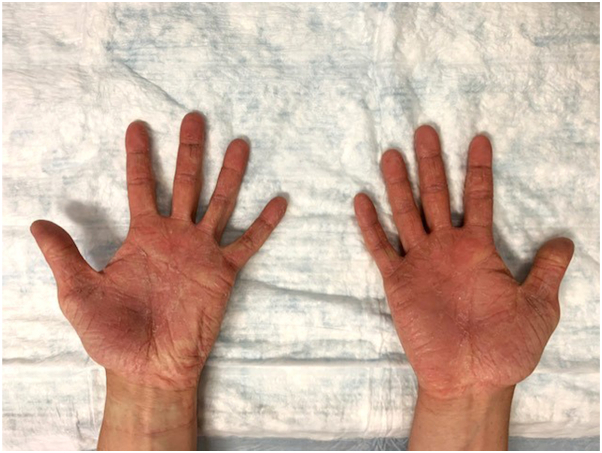
Bilateral palms with waxy hyperkeratotic surface and hyperlinearity with some transgrediens extension and mild erythematous border of volar wrists.

**Fig 2:**
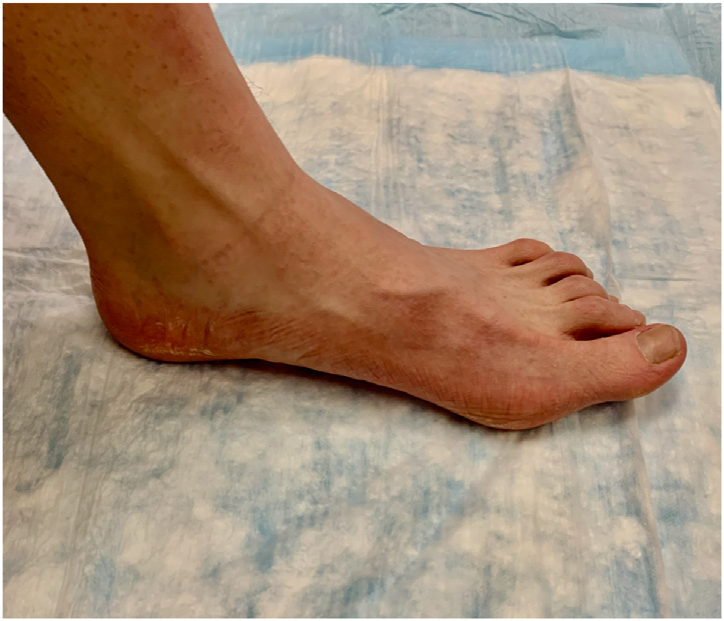
Waxy hyperkeratotic surface with some transgrediens hyperlinearity and mild erythematous border.

**Fig 3:**
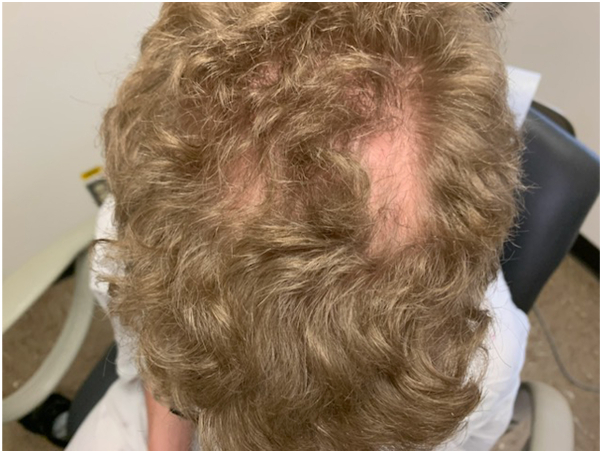
Well-demarcated patches of stable scarring alopecia on the parietal scalp without secondary features.

A 21-year-old man presented to the dermatology clinic with nonpruritic asymptomatic transgrediens waxy hyperlinear hyperkeratotic surfaces of his palms and soles (Figs 1 and 2). Upon further examination, 2 well-demarcated patches of scarring alopecia were noted on the parietal scalp (Fig 3). The patient’s nails appeared normal, and the rest of the examination was unremarkable. Upon review, the patient’s medical record revealed no hearing loss, abnormal dentition, or ocular disease. The patient denied having any other family members with a similar condition. He reported that he first noticed thickening and roughness of his plantar feet when he was 5 years old. Similar changes on his palms began at approximately 12 years of age. When asked about his scalp alopecia, the patient associated it with childhood trauma. However, the remembered event was mild and unlikely to produce scarring alopecia. Owing to his unique constellation of symptoms, medical genetics was consulted. Genetic sequencing identified a heterozygous deletion of exons 14 to 16 in the *TRPV3* gene located on chromosome 17p13. This variant of uncertain significance is expected to create a premature stop signal and result in an absent or disrupted protein product.

## Discussion

OS is clinically characterized by marked transgrediens palmoplantar keratoderma and periorificial keratotic plaques, which may be painful or pruritic. Most cases of OS have reported various hair abnormalities, including alopecia, which was consistent with the fingings of our physical examination. Additional variable phenotypic manifestations of OS may include deafness, corneal dysplasia, hyperkeratotic linear streaks, erythromelalgia, onychodystrophy, and digital constriction.^2^ These were not features of our patient’s presentation. We hypothesize that this mild presentation is a result of a loss-of-function rather than gain-of-function mutation.

Historically, there have been no targeted treatments for this rare disease. However, recently, there have been promising case reports demonstrating the efficacy of an epidermal growth factor receptor inhibitor.^5^, ^6^, ^7^ It is unclear if these therapies would be effective for our patient given his mutation status. Our patient’s palmoplantar keratoderma is well-managed with the use of topical therapies including urea and clobetasol. He reports often self-discontinuing therapy because he does not desire complete resolution of his palmar symptoms, which he finds advantageous for his profession as an aircraft mechanic.

## Conflicts of interest

None disclosed.
